# Application of tabu search-based Bayesian networks in exploring related factors of liver cirrhosis complicated with hepatic encephalopathy and disease identification

**DOI:** 10.1038/s41598-019-42791-w

**Published:** 2019-04-18

**Authors:** Zhuang Zhang, Jie Zhang, Zhen Wei, Hao Ren, Weimei Song, Jinhua Pan, Jinchun Liu, Yanbo Zhang, Lixia Qiu

**Affiliations:** 1 Department of Health Statistics, School of Public Health, Shanxi Medical University, No.56 XinJian South Road, Taiyuan, Shanxi 030001 China; 2 Department of Basis, Jinci College of Shanxi Medical University, No.2 Jinliao Road, Jinci Town, Jinyuan District, Taiyuan, Shanxi 030025 China; 3 Puruisheng (Beijing) Pharmaceutical Technology Development Co., Ltd., No. 188 South Fourth Ring Road, Fengtai District, Beijing, 100071 China; 4 Department of Gastroenterology, First Hospital of Shanxi Medical University, No.85 JieFang South Road, Taiyuan, Shanxi 030001 China

**Keywords:** Liver cirrhosis, Disorders of consciousness, Applied mathematics

## Abstract

This study aimed to explore the related factors and strengths of hepatic cirrhosis complicated with hepatic encephalopathy (HE) by multivariate logistic regression analysis and tabu search-based Bayesian networks (BNs), and to deduce the probability of HE in patients with cirrhosis under different conditions through BN reasoning. Multivariate logistic regression analysis indicated that electrolyte disorders, infections, poor spirits, hepatorenal syndrome, hepatic diabetes, prothrombin time, and total bilirubin are associated with HE. Inferences by BNs found that infection, electrolyte disorder and hepatorenal syndrome are closely related to HE. Those three variables are also related to each other, indicating that the occurrence of any of those three complications may induce the other two complications. When those three complications occur simultaneously, the probability of HE may reach 0.90 or more. The BN constructed by the tabu search algorithm can analyze not only how the correlative factors affect HE but also their interrelationships. Reasoning using BNs can describe how HE is induced on the basis of the order in which doctors acquire patient information, which is consistent with the sequential process of clinical diagnosis and treatment.

## Introduction

Hepatic encephalopathy (HE) is a very serious complication of liver disease. It is a comprehensive symptom of central nervous system dysfunction based on metabolic disorders, caused by acute or chronic severe liver function disorder, or abnormal portal-systematic circulation^1^. Most patients with cirrhosis have different degrees of HE at varying stages of the disease, the mortality rate of which is extremely high^2,3^. The 1-year survival rate of patients who have chronic liver disease complicated with HE is 0.42, and their 3-year survival rate is 0.23^4^. HE is divided into dominant and occult types^1^: dominant HE has obvious clinical manifestations, such as disturbance of consciousness, behavioral disorders, and coma^5^; whereas the clinical signs of occult HE are difficult to detect. Thus, prevention and early identification of HE could be a reasonable and effective way to reduce HE harm. At present, there is no “gold standard” for HE diagnosis. Clinicians can only conduct auxiliary diagnostic evaluation of HE from different angles such as liver function tests, imaging examinations, and neuropsychiatric abnormalities. Occult HE often has no clinical symptoms, and only through careful neuropsychological or neurophysiological examination can patients be found to have cognitive dysfunction. However, the neuropsychological testing process is cumbersome, can consume too much time, and is easily susceptible to the patient’s condition, educational level, and cultural background. Neurophysiological tests are inaccurate and require complex instruments, and their routine clinical applicability is relatively limited^6^. In short, the pathogenesis of HE is complex and diverse, and its clinical symptoms are variable and sometimes difficult to find. There are no clear diagnosis or treatment guides for it at home or abroad. Therefore, search methods for clinical diagnosis and treatment of patients with cirrhosis use patients’ clinical manifestations and factors that affect the occurrence of HE to identify HE, which has important clinical diagnostic value and is worthy of our research.

In recent years, with the development of hospital information technology, hospital information systems contain large amounts of patient and medical data such as patient sociodemographic characteristics, clinical symptoms, physiological and biochemical indicators, and auxiliary examinations, which have formed large datasets^7,8^. Full and reasonable use of these data is an important basis for current big data analysis. We can use traditional logistic regression on the big data generated during clinical diagnosis and treatment of patients with cirrhosis to establish a prediction model for the incidence of HE in patients with cirrhosis^9^, and finally make judgments based on clinical expertise. Logistic regression requires that the independent variables be independent^9^, but the clinical manifestations of HE and the factors affecting the occurrence of HE are not independent, and there may be interactions that form complex networks of relationships. Therefore, it is difficult for logistic regression to accurately reveal the potential overall information in the data, and it is impossible for logistic regression to infer which factors play a role in the occurrence and development of HE. In addition, any logistic regression model that is applied must predict the probability of developing HE when the state of each variable is known. In clinical practice, the factors used for model prediction may be missing: patients may conceal certain information, or some items may not be detected in clinical diagnosis, which could lead to the failure of logistic prediction.

Bayesian networks (BNs), proposed by Judea Pear in the 1980s, reflect the potential relationships and relationship strengths between factors by constructing directed acyclic graphs (DAGs) and conditional probability distribution tables^10^. BNs do not have strict requirements for statistical assumptions in the modeling process^11^. Although BNs do not extract variables’ main effects and interaction effects, they comprehensively reflect the complex relationships between variables according to the overall structure of the network and can accurately reveal potential overall information in the data^12^. BNs can use missing data to identify patients^13^ in a manner that reasonably reflects the sequentiality of clinical diagnosis and treatment^14,15^. The advantages of BNs in clinical applications have also been demonstrated in our previous studies. Because there were too many initial variables in this study’s initial cirrhosis data, some variables were only weakly related to the occurrence and development of HE. The direct use of a BN to establish the model leads to the selection of all variables, which is not conducive to accurate analysis. To simplify the network’s relationships and improve network performance, in this study we employed univariate and multivariate logistic regression analysis to screen for related factors of cirrhosis with HE^16^, and then constructed a BN to explore the related factors of cirrhosis complicated with HE, revealing the complex network relationships among related factors^17^. During the care process of patients with cirrhosis, we can continuously update the network probabilities according to the patient information obtained by doctors. We also adopt BNs to conduct risk reasoning about the probability of HE in patients with cirrhosis^18,19^.

## Materials and Methods

### Data collection

We collected information about patients with cirrhosis who were hospitalized in the Department of Gastroenterology, First Hospital of Shanxi Medical University from January 2006 to December 2015 and who had complete medical records. We consulted a large number of documents and carefully analyzed the cases’ structure and content and selected indicators that may be related to the progression of cirrhosis. Biochemical indicators were taken as the results of the first test within 24 hours after admission. After the data were sorted, 950 cases were effective, of which 68 were complicated by HE.

There are 23 factors in the survey: demographic characteristics (age, gender), lifestyle (smoking, drinking), past medical history (e.g., hypertension and hepatic diabetes), clinical manifestations (infection, electrolyte disorder, hepatorenal syndrome, spontaneous peritonitis, upper gastrointestinal bleeding, poor spirits, liver disease face, spider nevus, liver palm, jaundice, abdominal varicose veins, splenomegaly, hepatomegaly, ascites), and biochemical indicators (serum albumin [g/L], serum total bilirubin [mmol/L], and prothrombin time [s]). All of this study’s methods were carried out in accordance with relevant guidelines and regulations.

### Ethical statements

This study was approved by the First Hospital of Shanxi Medical University Ethics Committee. Informed consent was signed by all study participants or their agents. The present study was conducted according to the Declaration of Helsinki.

### Quality control

This study’s investigators were clinicians and graduate students. Before the start of the survey, the clinicians instructed the graduate students to familiarize themselves with clinical records and to determine the location of the information to be collected in the medical records. Finally, the clinicians normalized the course of looking up medical records. Data entry was performed by double entry, and a person reviewed both sets of entries. If anomalous values were discovered during the review process, the original case was extracted and the erroneous information was corrected.

### Bayesian networks

BNs are increasingly popular data-mining techniques that are used with categorical data and effective theoretical models to represent uncertain knowledge and reasoning^20^. As a reasoning method that employs probabilistic uncertainty, BNs have served in intelligent systems and have been successfully applied in medical diagnosis, expert systems, statistical decision making, learning, and prediction^21^.

BNs represent a set of random variables and their conditional interdependencies via a Directed Acyclic Graph (DAG)^22^. The DAG’s nodes represent random variables U = {${{\rm{X}}}_{{\rm{i}}}$, …, ${{\rm{X}}}_{{\rm{n}}}$}, and the directed edges E represent the probabilistic dependency relations between variables^23,24^. If there is a directional arc from ${X}_{{\rm{i}}}$ to ${X}_{{\rm{j}}}$, we can informally regard that ${X}_{{\rm{i}}}$ causes ${X}_{{\rm{j}}}$, so that ${X}_{{\rm{i}}}$ and ${X}_{{\rm{j}}}$ are usually said to be parent and child, respectively. Each node has a conditional probability distribution table that represents the conditional probability distribution of each node in the state of a given parent node. The BN is a representation of the joint probability distribution of a set of random variables *X* = $\{{X}_{1},\ldots ,{X}_{n}\}$, so a probability expression can be obtained:

1 $$P({X}_{1},\ldots ,{X}_{n})=P({X}_{1})P({X}_{2}|{X}_{1})\ldots P({X}_{n}|{X}_{1},{X}_{2},\ldots ,{X}_{n-1})=\prod _{1}^{n}\,P({X}_{i}|\pi ({X}_{i}).$$

$\pi ({X}_{i})\,\,$is the collection of the parent of ${X}_{i}$, $\,\pi ({X}_{i})$ ⊆ $\{{X}_{1},\ldots ,{X}_{i-1}\}$.

The first step of constructing a BN is to determine the nodes. To avoid including too many nodes and introducing excessive complexity to the network structure, we should initially filter the nodes in combination with prior expert knowledge or traditional statistical methods. Then, we should find the optimal model based on the tabu search algorithm. Finally, on the basis of structural learning, we should calculate the network node conditions using the maximum likelihood estimation method.

### Tabu search algorithm

The tabu search algorithm is a sub-heuristic algorithm that simulates human memory function. It has the characteristics of few parameters, simple structure, and robust global optimization ability. For a given current network structure, the algorithm uses three operations to generate neighborhoods without generating a network loop: add edges, reduce edges, and reverse edges, and then it searches for a local optimal solution in the neighborhood and puts it into a tabu table. The tabu table is then used to search for the local optimal solution, so that the next search is as far as possible from the current one to avoid duplication of the search process. In conjunction with the use of contempt criteria to amnesty some of the optimal solutions in the tabu table, the tabu restrictions are ignored. When these two steps iterate and search in a circular way, it results in a taboo table that is updated constantly, and ultimately, we obtain the global optimal solution^25^.

### Definitions

(1) Cirrhosis: comply with the diagnostic criteria jointly revised by the Chinese Medical Association’s Infectious Diseases and Parasitic Diseases Branch and the Hepatology Branch in 2000^1^; (2) HE: refers to the “definition of HE definition, diagnosis, and quantification” standard issued by the 11th World Conference on Gastroenterology Work Group in March 2003, with the diagnosis mainly based on cirrhosis history, neurological abnormalities, liver function tests, image examination, and other auxiliary examination, excluding nerve abnormalities caused by other diseases^26^; (3) Hypertension: according to the World Health Organization (WHO) standards, systolic blood pressure SBP ≥ 140 mmHg and/or diastolic blood pressure DBP ≥ 90 mmHg, or having a previous history of hypertension and currently taking antihypertensive drugs^27^; (5) Diabetes mellitus: fasting blood glucose (FBG) ≥ 7.0 mmol/L or 2-hour postprandial plasma glucose ≥ 11.1 mmol/L or reporting diagnosed diabetes mellitus^28^; (6) Smoker: smoking ≥ 1 cigarette daily on average over the previous 6 months; (7) Drinker: drinking at least once per week regularly for 6 consecutive months with average intake quantity of more than 50 g^29^.

### Establish database and statistical analysis

In our study, we used Epidata to build a database and enter data. Statistical Analysis System (SAS) version 9.2 software (SAS Institute Inc., Cary, NC, USA) was used for data pretreatment. Descriptive statistics and logistic regression analysis of the data was performed with SPSS Version 22 (IBM Corp., Armonk, NY, USA). Significance for all statistical tests was set a priori at P < 0.05, and all P values were two-tailed. Weka 3.8.0 (Waikato Environment for Knowledge Analysis; the University of Waikato, New Zealand) was used for structural learning of BNs and parameter estimation of BNs using the maximum likelihood estimation method. The BNs’ topology and conditional probability distribution tables were drawn using Netica (Norsys Software Corp., Vancouver, BC, Canada).

## Results

### Characteristics of the study population

**Figure 1:**
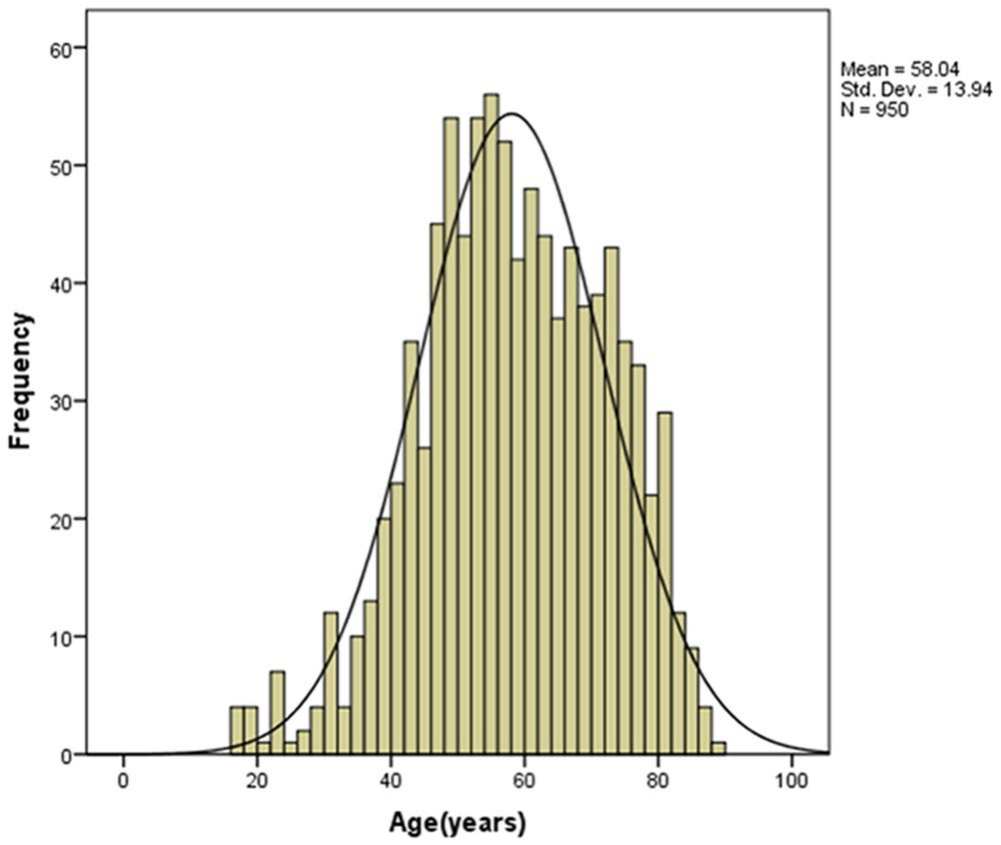
Age distribution characteristics of 950 patients with cirrhosis. The figure was plotted using Spss22.0 (https://www.ibm.com).

The total enrollment was 950 patients with liver cirrhosis, including 508 male (53.5%) and 442 female (46.5%) individuals ranging 17–88 years old, with a negatively skewed distribution. The overall median age was 58.0 years (QR = 20.3). Among the subjects, 156, 357, and 437 were aged <45, 45–59, and ≥60 years, respectively (Fig. 1).

### Univariate analysis

There were 68 cases of HE among the 950 total patients with cirrhosis. The average prevalence of HE was 7.16%. To investigate the prevalence of concurrent HE, The patients were grouped by 23 factors such as demographic information, clinical comorbidities, clinical manifestations, and biochemical parameters. Suppl. Table S1 shows the factors and their assignment. Among the factors, 15 of them were statistically significant in patients with cirrhosis complicated by HE: infection, electrolyte disorder, hepatorenal syndrome, spontaneous peritonitis, hepatic diabetes, poor spirits, liver disease face, spider nevus, liver palm, jaundice, abdominal varicose veins, hepatomegaly, albumin, increased total bilirubin, and prothrombin time prolongation (all Ps < 0.05). Suppl. Tables S2, S3, and S4 represent differences in the prevalence of HE among patients with cirrhosis who had different characteristics.

### Multivariate analysis

The multivariate logistic regression was accompanied by stepwise variable selection to analyze the 15 factors related to HE. The variable selection and rejection criteria were 0.05 and 0.1, respectively.

The final regression model contained seven factors: infection, electrolyte disorders, hepatorenal syndrome, hepatic diabetes, poor spirits, increased total bilirubin, and prothrombin time prolongation. Among them, electrolyte disorders (RR = 6.861), hepatorenal syndrome (RR = 3.467), and infection (RR = 3.021) were the major risk factors for HE in patients with cirrhosis. The relationships between hepatic diabetes, poor spirits, increased total bilirubin, prothrombin time prolongation, and HE were relatively weak, with RR values ranging 2.1–2.7 (Table 1).

**Table 1:** Multivariate logistic regression analyses of related factors of hepatic encephalopathy.

Factors	*β*	SE	*Waldχ* ^*2*^	*P*	*RR*(*95% CI*)
Electrolyte disorder	1.926	0.369	27.173	<0.001	6.861(3.326~14.154)
Hepatorenal syndrome	1.243	0.638	3.799	0.051	3.467(0.993~12.101)
Infection	1.106	0.331	11.160	0.001	3.021(1.579~5.778)
Poor spirit	1.001	0.292	11.755	0.001	2.721(1.535~4.822)
Hepatic diabetes	0.871	0.340	6.558	0.010	2.390(1.227~4.656)
Prothrombin time (s)	0.830	0.267	9.691	0.002	2.293(1.360~3.866)
Total bilirubin (mmol/L)	0.703	0.172	16.720	<0.001	2.020(1.442~2.829)

αα_in_ = 0.05, α_out_ = 0.10.

Logistic regression was used to screen possible factors related to HE and evaluate their associated risk intensity. However, logistic regression analysis requires independence among variables and cannot account for the correlation between independent variables. The model can only explain the direct relationship between these factors and HE. In terms of information about current status, the results showed only factors associated with HE and did not reflect the complex relationship between the factors, diseases, and outcomes.

### Bayesian network model

BNs are DAGs, which can help to describe the relationship between HE and these possible factors.

#### Construct Bayesian network structure

**Figure 2:**
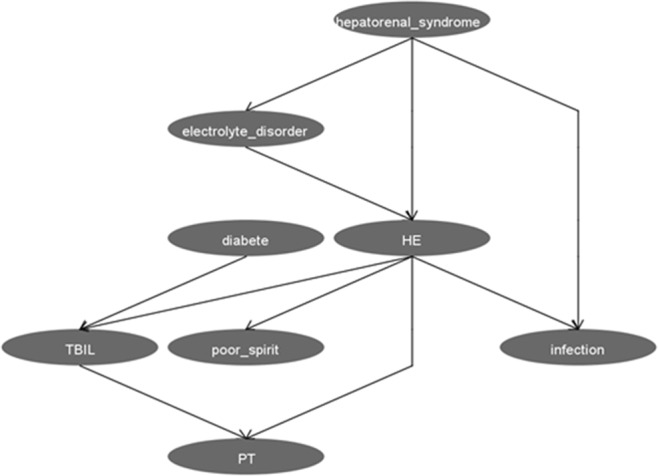
The Bayesian Network model for related factors of HE. The figure was plotted using Weka3.8.0 (http://weka.wikispaces.com/).

The algorithm for constructing a BN structure is the tabu search algorithm, which is achieved by the Weka tabu search module. The path is Weka.classifiers.bayes.net.search.local.TabuSearch. The BN model of HE-related factors consists of 8 nodes and 10 directed edges. Each node represents HE, electrolyte disorders, infections, poor spirits, hepatorenal syndrome, diabetes, prothrombin time, and total bilirubin (Fig. 2).

Figure 2 shows that various factors establish links with HE through complex relationships: hepatorenal syndrome, electrolyte disorders, infections, poor spirits, total bilirubin, and prothrombin time are directly related to HE, and hepatic diabetes is indirectly associated with total bilirubin. In addition, hepatorenal syndrome is indirectly linked with HE via its association with electrolyte disorders and infections.

#### Bayesian network parameter learning

**Figure 3:**
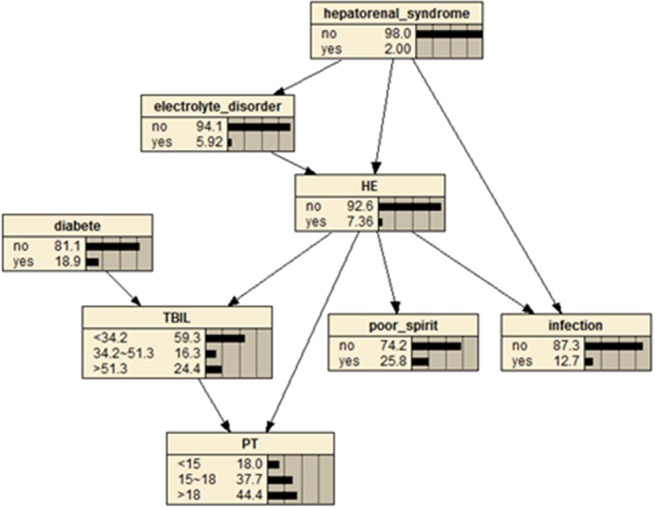
Prior probability of each node in the Bayesian Network. The figure was plotted using Netica (www.norsys.com).

Based on the BN structure, we used the maximum likelihood estimation method to estimate the probability of each node in the network conditions. Figure 3 shows the a priori probability of each node in the network. The probability of HE in patients with liver cirrhosis was 0.0736. As depicted in Table 2, if patients had hepatorenal syndrome but not electrolyte disorder, the incidence of HE was reduced to 0.048; if only hepatorenal syndrome occurred, the incidence of HE was 0.300; if only electrolyte disorder, the probability of concurrent HE was 0.383; and when hepatorenal syndrome and electrolyte disorder occurred together, The incidence of HE was 0.667. When the above two phenomena occur together, we should pay great attention to prevent the occurrence of HE.

**Table 2:** The conditional probability distribution table with hepatorenal syndrome and electrolyte disorder as parent nodes.

Parent nodes		HE	
Hepatorenal syndrome	Electrolyte disorder	YES	NO
YES	YES	0.667	0.333
YES	NO	0.300	0.700
NO	YES	0.383	0.617
NO	NO	0.048	0.952

#### Bayesian network evaluation

**Figure 4:**
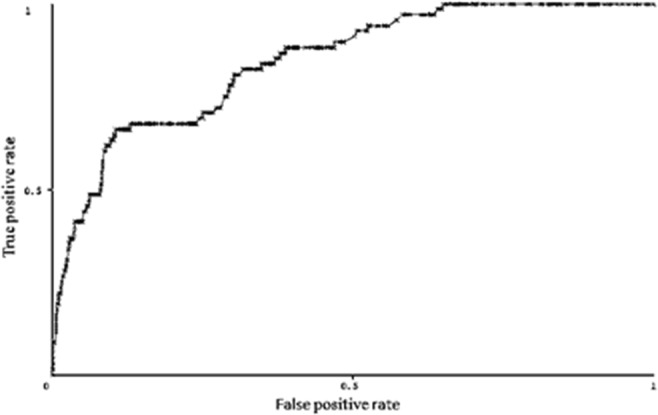
ROC curve of the Bayesian network of hepatic encephalopathy. The figure was plotted using Weka3.8.0 (http://weka.wikispaces.com/).

We used the area under the receiver operating characteristic (ROC) curve as the evaluation index. Figure 4 shows that the network’s area under the ROC curve is 0.843. Thus, the network has high sensitivity and specificity and reflects the dependence among nodes.

### Reasoning model

BN reasoning refers to calculating the probability of an event in a given network structure, given the available evidence. Its biggest advantage is automatic updating of network probabilities according to different degrees of information mastery.

According to the patients’ visiting process and the order in which information was obtained by the doctors, we used the BN to predict which patients would have concurrent HE. When a patient with cirrhosis does not attend a clinical examination, the likelihood of HE is 0.0736 by a priori information about the BN. If a clinical examination reveals that the patient is in poor spirits and has hepatic diabetes, then the BN shows that the likelihood that the patient has HE is 0.155 (Suppl. Fig. S1). If the patient’s total bilirubin is greater than 51.3 mmol/L, or prothrombin time is longer than 18s, then the probability of concurrent HE increases to 0.341 (Suppl. Fig. S2). If the patient has any of the factors of infection, electrolyte disturbance, or hepatorenal syndrome, the probability of concurrent HE is more than 0.60: 0.615, 0.834, and 0.867, respectively (Suppl. Fig. S3, S4, and S5). Thus, infections, electrolyte disorders, and hepatorenal syndrome are closely associated with HE: if those three conditions are all present, the likelihood of concurrent HE is 0.968 (Suppl. Fig. S6).

## Discussion

In the medical field, prediction of disease risk requires the establishment of a statistical model of risk factors and diseases and prediction of the probability of disease according to levels of multiple risk factors^15^. Our study’s data are cross-sectional survey data that contain both causal and outcome variables of HE. Thus, it is not reasonable to use them to predict the risk of HE with a logistic regression model. Moreover, the use of a logistic regression model requires knowing all variables that predict disease risk^9^. From the perspective of economic rationality, doctors cannot require patients to undergo all tests that can exclude the disease at once; thus, logistic regression does not conform to clinical practice. BN reasoning can handle incomplete datasets and probabilistic reasoning based on the amount of information available to the patient. With increased amounts of patients’ information collected, the network probabilities are updated constantly, and the likelihood of HE among patients with cirrhosis is evaluated according to different symptoms and signs. Therefore, the BN method is more suited to the clinical process of sequential diagnosis and treatment^11,12,15^.

All clinical factors contribute to the occurrence and development of HE through complex network relationships^5,30^. In this study, we used logistic regression with univariate and multivariate models to screen the main correlates of cirrhosis complicated with HE, and the BN structure was constructed to estimate the conditional probability of each node^31–33^. The area under the ROC curve was 0.843, which indicated high sensitivity and specificity of the network construction of the tabu search algorithm. The BN can reflect the relationship between the various nodes so as to provide a basis for evaluation of influencing factors on HE.

Our results show that hepatorenal syndrome, electrolyte disorders, poor spirits, infections, prothrombin time, and total bilirubin are directly associated with HE. Hepatogenic diabetes and HE are linked through total bilirubin^34^ (Fig. 2). Studies have suggested that impaired glucose tolerance is a key determinant of the prognosis of patients with cirrhosis. Abnormal blood glucose in patients with cirrhosis increases the risk of various complications and affects patients’ prognosis. Therefore, hepatogenic diabetes is likely to cause HE indirectly by aggravating hepatic impairment^5^. Hepatorenal syndrome is also related to electrolyte disorder and infection. Mathurin et al. showed that the risk coefficient of HE increased with decreased serum sodium and serum cholinesterase levels and increased blood urea nitrogen levels in patients with liver cirrhosis and renal insufficiency^35^. A large number of patients suffer from anorexia and ascites among those with liver cirrhosis. Salt intake should be strictly controlled as part of treatment. However, long-term administration of high-dose diuretics can lead to long-term low sodium, chloride, and potassium status. Severe imbalances disrupt electrolyte and acid-base balance in cells’ internal and external fluids^36,37^, which seriously affects their metabolism and physiological functions. This eventually leads to brain edema and induces HE^38^. Some studies have shown that infection is related to HE^39,40^; however, whether or not there is a causal relationship is still unclear. It is generally believed that when an organism becomes infected, catabolism increases; ammonia production increases; and liver, brain, kidney, and other solid organs are damaged. Hypoxia or hyperthermia increase the brain’s sensitivity to ammonia toxicity and induce or aggravate HE^38,41^. To a certain extent, increased total bilirubin and prolonged prothrombin time may reflect the deterioration of liver function, and severe cases of liver cirrhosis may be complicated by HE^34^.

The BN was used to induce the probability of HE in patients with cirrhosis. When a patient with cirrhosis has not been clinically examined, the relevant symptoms and indicators are unknown. The BN showed that the probability of concurrent HE in such cases was 0.0736. If the clinical examination found that the patient was apathetic and had hepatic diabetes, the likelihood of concurrent HE increased to 0.155; if the patient’s total bilirubin was greater than 51.3 mmol/L and prothrombin time was longer than 18 s, the probability of concurrent HE increased to 0.341; if the patient showed any of the factors of infection, electrolyte disorders, or hepatorenal syndrome, the probability of concurrent HE exceeded 0.60 (i.e., 0.615, 0.834, and 0.867, respectively). Infection, electrolyte disorders, and hepatorenal syndrome are closely related to HE: if the patient had all three conditions, the probability of concurrent HE was 0.968. The results show that the BN constructed by the tabu search algorithm has a flexible reasoning mechanism, which is helpful for disease identification and diagnosis. It has important clinical diagnostic value and could serve as an effective method to reduce harm from HE.

The complex network of relationships between HE-related factors revealed by the BN is in line with clinical practice. By constructing the BN model, the clinical manifestations of patients with cirrhosis and the factors affecting their probability of occurrence of HE are used to identify the cirrhosis with HE, and the network probabilities can be updated as the doctor receives increasing amounts of patient information. Although this study lists only some of the key issues, it shows that compared with a logistic probability prediction model, the BN is more in line with the sequential process of clinical diagnosis and treatment. BNs have other advantages, including their ability to combine prior information and sample information. Subjective bias caused by the application of prior information and bias caused by using only sample information can be avoided^42^. BNs are also suitable for datasets with a variety of uncertain information and excessive numbers of variables^11^: they facilitate easy understanding and explanation through visual images of the results. However, BNs also have shortcomings: for example, the edges between variables only represent probability dependencies and do not represent causal relationships. Therefore, we cannot perform causal analysis from only the BN’s directed edges. It is necessary to combine various aspects of professional knowledge to determine causality^43^. The data in this study are unbalanced, which may cause incorrect classification. In future research, we intend to deal with the impact of data imbalance on the classification model at the data and algorithm levels and hope to achieve better results. For example, we can expand the scope of data collection to increase the number of HE-positive cases and continuously verify and improve the accuracy and applicability of model classification identification. In addition, this study is based on a cross-sectional survey; thus, the BN can only reveal the relevant factors related to the occurrence of HE, and the causal relationship needs further verification. Only in a BN built making use of causal relationships do the directed edges between the variables represent causal relationships^44^.

## Supplementary information

Supplementary information

Dataset

## Data Availability

All data generated or analyzed during this study are included in its Supplementary Information files.
