# Supplementary material for: Application of tabu search-based Bayesian networks in exploring related factors of liver cirrhosis complicated with hepatic encephalopathy and disease identification
Source: Sci Rep. 2019 Apr 18;9:6251. doi: 10.1038/s41598-019-42791-w (PMC6472503; doi:10.1038/s41598-019-42791-w)
Supplement: Supplementary file 1 — Supplementary information [file 41598_2019_42791_MOESM1_ESM.docx]

**Application of** **tabu search-based Bayesian Networks in exploring related factors of liver cirrhosis complicated with hepatic encephalopathy and disease identification**

Zhuang Zhang^1+^,Jie Zhang^2+^,Zhen Wei^3+^,Hao Ren^1^ ,Weimei Song^1^,Jinhua Pan^1^,Jinchun Liu^4^, Yanbo Zhang^1^, Lixia Qiu^1,*^

1. Department of Health Statistics, School of Public Health, Shanxi Medical University, No.56 XinJian South Road, Taiyuan, Shanxi 030001, China

2. Department of Basis, Jinci College of Shanxi Medical University, No.2 Jinliao Road, Jinci Town, Jinyuan District, Taiyuan, Shanxi 030025, China

3. Puruisheng (Beijing) Pharmaceutical Technology Development Co., Ltd., No. 188 South Fourth Ring Road, Fengtai District, Beijing100071, China

4. Department of Gastroenterology, First Hospital of Shanxi Medical University, No.85 JieFang South Road, Taiyuan, Shanxi 030001, China

+ These authors contributed equally to this work

*Correspondence and requests for materials should be addressed to L.X.Q(E-mail: qlx_1126@163.com;Phone:+86-13453189351)

| Factors | Assignment |
| --- | --- |
| Gender($x_{1}$) | Male^*^=1;Female=2 |
| Age($x_{2}$) | <45^*^=1;45～=2;60～=3 |
| Smoking($x_{3}$) | NO^*^ =0;YES =1 |
| Drinking($x_{4}$) | NO^*^ =0;YES =1 |
| Infection($x_{5}$) | NO^*^ =0;YES =1 |
| Electrolyte Disorder($x_{6}$) | NO^*^ =0;YES =1 |
| Hepatorenal Syndrome($x_{7}$) | NO^*^ =0;YES =1 |
| Spontaneous Peritonitis($x_{8}$) | NO^*^ =0;YES =1 |
| Upper Gastrointestinal Bleeding($x_{9}$) | NO^*^ =0;YES =1 |
| Hypertension($x_{10}$) | NO^*^ =0;YES =1 |
| Hepatic diabete($x_{11}$) | NO^*^ =0;YES =1 |
| Poor spirit($x_{12}$) | NO^*^ =0;YES =1 |
| Liver disease face($x_{13}$) | NO^*^ =0;YES =1 |
| Spider nevus($x_{14}$) | NO^*^ =0;YES =1 |
| Jaundice($x_{15}$) | NO^*^ =0;YES =1 |
| Liver palm($x_{16}$) | NO^*^ =0;YES =1 |
| Abdominal Varicose veins($x_{17}$) | NO^*^ =0;YES =1 |
| Splenomegaly($x_{18}$) | NO^*^ =0;YES =1 |
| Hepatomegaly($x_{19}$) | NO^*^ =0;YES =1 |
| Ascites($x_{20}$) | NO^*^ =0;YES =1 |
| Albumin(g/L) ($x_{21}$) | >35^*^ =1;28~35=2;<28=3 |
| TBIL(mmol/L) ($x_{22}$) | <34.2^*^ =1;34.2～51.3=2;>51.3=3 |
| PT(s) ($x_{23}$) | <15^*^ =1;15~18=2; <18=3 |
| HE(y) | NO^*^=1;YES =2 |

^* Reference standard^

Supplementary Table S1 Factors and assignment

| Factors | cases | HE | Prevalence (%) | *χ^2^* | *P* |
| --- | --- | --- | --- | --- | --- |
| **Gender** | | | | | |
| Male | 508 | 39 | 7.7 | 0.442 | 0.506 |
| Famale | 442 | 29 | 6.6 |  |  |
| **Age** | | | | | |
| <45 | 156 | 7 | 4.5 | 0.703 | 0.402 |
| 45～ | 357 | 29 | 8.1 |  |  |
| 60～ | 437 | 32 | 7.3 |  |  |

### ^α=0.05^

### Supplementary Table S2 Comparison of differences in prevalence of HE among different age groups and gender

| Factors | cases | HE | Prevalence (%) | *χ^2^* | *P* |
| --- | --- | --- | --- | --- | --- |
| **Smoking** | | | | | |
| NO | 320 | 25 | 7.8 | 0.311 | 0.577 |
| YES | 630 | 43 | 6.8 |  |  |
| **Drinking** | | | | | |
| NO | 651 | 40 | 6.1 | 3.153 | 0.076 |
| YES | 299 | 28 | 9.4 |  |  |

### ^α=0.05^

### Supplementary Table S3 Comparison of differences in prevalence of HE among different lifestyles

| Factors | cases | HE | Prevalence (%) | *χ^2^* | *P* |
| --- | --- | --- | --- | --- | --- |
| **Infection** | | | | | |
| NO | 831 | 45 | 5.4 | 26.477 | <0.001 |
| YES | 119 | 23 | 19.3 |  |  |
| **Electrolyte Disorder** | | | | | |
| NO | 895 | 44 | 4.9 | 74.867 | <0.001 |
| YES | 55 | 24 | 43.6 |  |  |
| **Hepatorenal Syndrome** | | | | | |
| NO | 932 | 59 | 6.3 | 30.209 | <0.001 |
| YES | 18 | 9 | 50.0 |  |  |
| **Spontaneous Peritonitis** | | | | | |
| NO | 901 | 55 | 6.1 | 23.687 | <0.001 |
| YES | 49 | 13 | 26.5 |  |  |
| **Upper Gastrointestinal Bleeding** | | | | | |
| NO | 504 | 34 | 6.7 | 0.237 | 0.626 |
| YES | 446 | 34 | 7.6 |  |  |
| **Hypertension** | | | | | |
| NO | 767 | 57 | 7.4 | 0.447 | 0.504 |
| YES | 183 | 11 | 6 |  |  |
| **Hepatic diabete** | | | | | |
| NO | 771 | 49 | 6.4 | 3.880 | 0.049 |
| YES | 179 | 19 | 10.6 |  |  |
| **Poor spirit** | | | | | |
| NO | 706 | 31 | 4.4 | 28.152 | <0.001 |
| YES | 244 | 37 | 15.2 |  |  |
| **Liver disease face** | | | | | |
| NO | 845 | 54 | 6.4 | 6.460 | 0.011 |
| YES | 105 | 14 | 13.3 |  |  |
| **Spider nevus** | | | | | |
| NO | 826 | 52 | 6.3 | 6.776 | 0.009 |
| YES | 124 | 16 | 12.9 |  |  |
| **Jaundice** | | | | | |
| NO | 634 | 28 | 4.4 | 19.804 | <0.001 |
| YES | 316 | 40 | 12.7 |  |  |
| **Liver palm** | | | | | |
| NO | 798 | 49 | 6.1 | 7.448 | 0.006 |
| YES | 152 | 19 | 12.5 |  |  |
| **Abdominal Varicose veins** | | | | | |
| NO | 894 | 59 | 6.6 | 6.603 | 0.010 |
| YES | 56 | 9 | 16.1 |  |  |
| **Splenomegaly** | | | | | |
| NO | 216 | 12 | 5.6 | 1.071 | 0.301 |
| YES | 734 | 56 | 7.6 |  |  |
| **Hepatomegaly** | | | | | |
| NO | 723 | 42 | 5.8 | 7.984 | 0.005 |
| YES | 227 | 26 | 11.5 |  |  |
| **Ascites** | | | | | |
| NO | 368 | 20 | 5.4 | 2.647 | 0.104 |
| YES | 582 | 48 | 8.2 |  |  |
| **Albumin(g/L)** | | | | | |
| >35 | 337 | 12 | 3.6 | 14.114 | <0.001 |
| 28~35 | 390 | 29 | 7.4 |  |  |
| <28 | 223 | 27 | 12.1 |  |  |
| **TBIL(mmol/L)** | | | | | |
| <34.2 | 565 | 17 | 3 | 42.558 | <0.001 |
| 34.2~51.3 | 155 | 10 | 6.5 |  |  |
| >51.3 | 230 | 41 | 17.8 |  |  |
| **PT(s)** | | | | | |
| <15 | 167 | 2 | 1.2 | 24.618 | <0.001 |
| 15~18 | 358 | 14 | 3.9 |  |  |
| >18 | 425 | 52 | 12.2 |  |  |

### ^α=0.05^

### Supplementary Table S4 Comparison of differences in prevalence of HE among different demographic characteristics clinical features

###
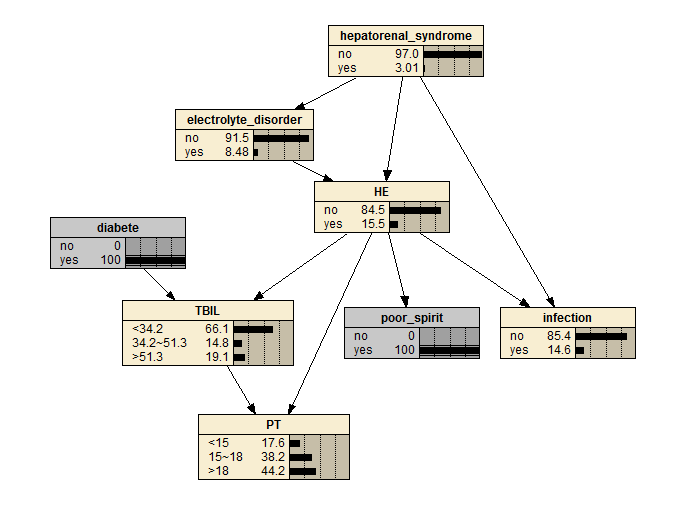


Supplementary Figure S1.The Bayesian network I under known evidence variables

###
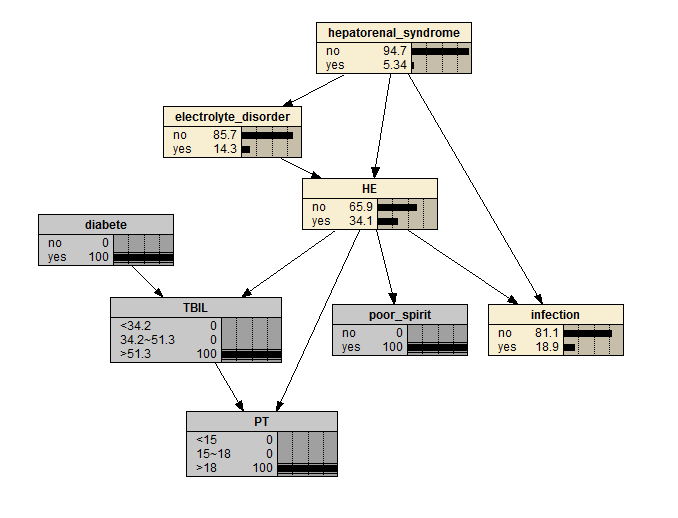


Supplementary Figure S2.The Bayesian network II under known evidence variables

###
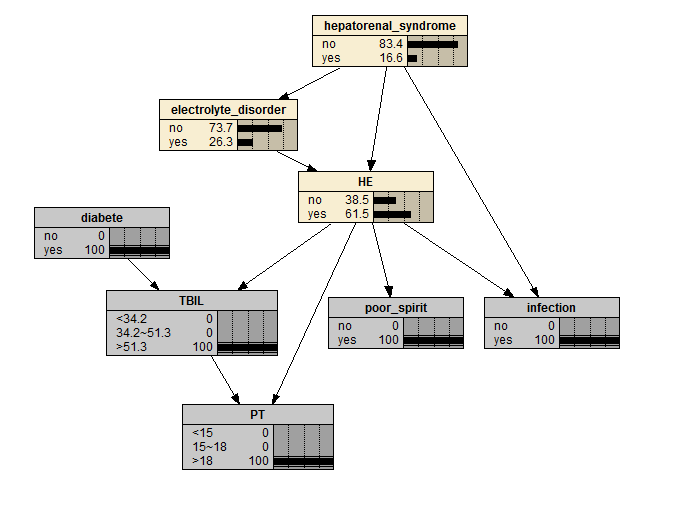


### Supplementary Figure S3 . The Bayesian network III under known evidence variables

###
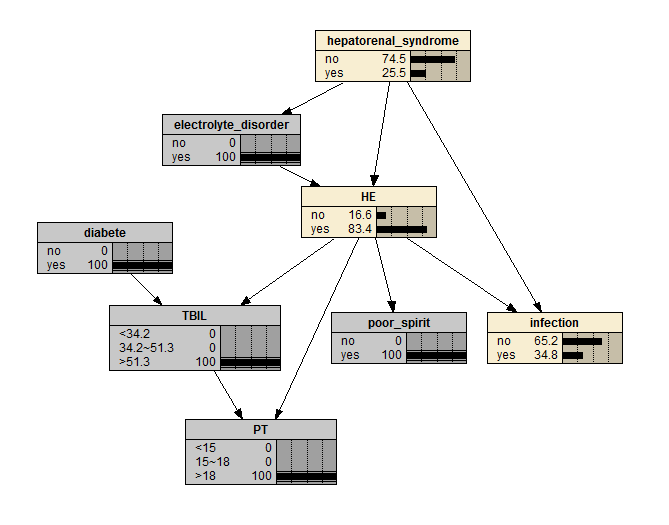


Supplementary Figure S4. The Bayesian network IV under known evidence variables

###
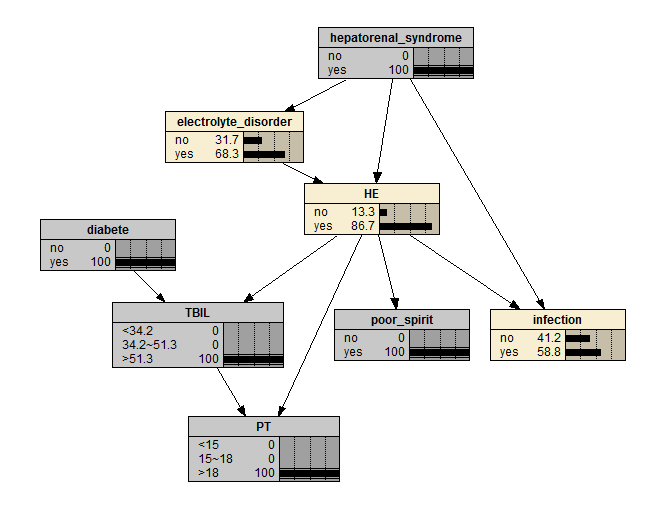


Supplementary Figure S5. The Bayesian network V under known evidence variables

###
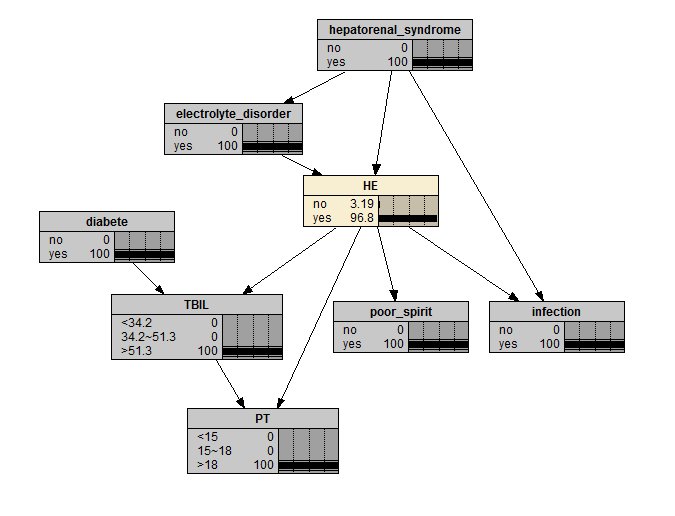


Supplementary Figure S6. The Bayesian network VI under known evidence variables
